# Concurrent brain responses to separate auditory and visual targets

**DOI:** 10.1152/jn.01050.2014

**Published:** 2015-06-18

**Authors:** Paola Finoia, Daniel J. Mitchell, Olaf Hauk, Christian Beste, Vittorio Pizzella, John Duncan

**Affiliations:** ^1^MRC Cognition and Brain Sciences Unit, Cambridge, United Kingdom;; ^2^Cognitive Neurophysiology, Department of Child and Adolescent Psychiatry, Universitätsklinikum Carl Gustav Carus an der Technischen Universität Dresden, Dresden, Germany;; ^3^Institute for Advanced Biomedical Technologies-I.T.A.B., University of Chieti and Pescara “G. D'Annunzio,” Chieti, Italy; and; ^4^Department of Experimental Psychology, University of Oxford, Oxford, United Kingdom

**Keywords:** crossmodal, attentional blink, fMRI, EEG, MEG

## Abstract

In the attentional blink, a target event (T1) strongly interferes with perception of a second target (T2) presented within a few hundred milliseconds. Concurrently, the brain's electromagnetic response to the second target is suppressed, especially a late negative-positive EEG complex including the traditional P3 wave. An influential theory proposes that conscious perception requires access to a distributed, frontoparietal global workspace, explaining the attentional blink by strong mutual inhibition between concurrent workspace representations. Often, however, the attentional blink is reduced or eliminated for targets in different sensory modalities, suggesting a limit to such global inhibition. Using functional magnetic resonance imaging, we confirm that visual and auditory targets produce similar, distributed patterns of frontoparietal activity. In an attentional blink EEG/MEG design, however, an auditory T1 and visual T2 are identified without mutual interference, with largely preserved electromagnetic responses to T2. The results suggest parallel brain responses to target events in different sensory modalities.

an enduring question is the neural basis for limits in divided attention, manifest in impaired performance when processing simultaneous or closely successive events (Broadbent 1958; Kahneman 1973; Marois and Ivanoff 2005; Pashler 1994). Such limits are effectively tested by presenting two target events, T1 and T2, at varying time intervals and recording performance in target detection or identification. When targets occur very close in time, performance on T2 is impaired, but recovers gradually as temporal separation increases. This phenomenon, called the attentional blink, is considered a key property of attentional resource limitation (Chun and Potter 1995; Raymond et al. 1992).

In vision, the neural basis for the attentional blink has been studied using event-related potentials (ERPs), comparing trials where T2 is correctly detected or identified (“seen” trials) to trials where T2 is missed (“unseen”). The major difference occurs in a late N2-P3 complex, severely attenuated or eliminated in the unseen case (Sergent et al. 2005; Vogel et al. 1998). This N2-P3 complex likely arises in widespread neural generators, including multiple frontal and parietal sources (Bledowski et al. 2004; Sergent et al. 2005), matching extensive frontoparietal activity for perceived targets of many kinds revealed by functional magnetic resonance imaging (fMRI) (Beck et al. 2001; Hampshire et al. 2010; Hon et al. 2006; Jiang et al. 2000; Marois et al. 2004). An influential theory proposes that this frontoparietal network constitutes a global workspace that broadcasts information widely between different brain systems and enables consciousness (Dehaene et al. 2003; Sergent and Dehaene 2004). Mutual inhibition within this network severely restricts the number of simultaneous events that can be processed (Sergent and Dehaene 2004) or operations that can be carried out (Raffone et al. 2014), explaining widespread limitations on divided attention and concurrent awareness including the attentional blink. This proposal is consistent with fMRI (e.g., Marois and Ivanoff 2005) and single-unit (Kadohisa et al. 2013; Watanabe and Funahashi 2014) data linking diverse aspects of restricted attentional capacity to competitive frontoparietal activity.

On this view, all targets should show mutual interference, irrespective of their nature or origin. In this regard, an intriguing puzzle concerns targets in different sensory modalities. Sometimes, in accord with the global workspace view, a blink occurs between targets in different modalities (e.g., Soto-Faraco et al. 2002), especially if T1 requires a speeded response (e.g., Jolicoeur 1999a; Jolicoeur 1999b), or if tasks for the two targets are very different, calling for a large change of mental set between T1 and T2 (e.g., Arnell and Jolicoeur 1999; Dell'Acqua et al. 2001). With unspeeded responses and similar T1/T2 tasks, however, many studies show the blink to be greatly reduced or eliminated with targets in different modalties (Arnell and Jenkins 2004; Arnell and Larson 2002; Duncan et al. 1997; Hein et al. 2006; Martens et al. 2010; Soto-Faraco and Spence 2002; Van der Burg et al. 2013; Van der Burg et al. 2007). These results suggest that, if behavior requires access to a global neuronal workspace, this workspace must be less limited than usually supposed. In line with well-known effects of task similarity in divided attention (Allport et al. 1972; Treisman and Davies 1973), mutual inhibition within the global workspace may depend on similarity between competing events.

Here we pursued this case of weak or absent attentional blink for unspeeded target identification in different sensory modalities. We tested the prediction that, when targets are identified without mutual interference, N2-P3 responses to both should be preserved. Using fMRI, we confirmed that targets in different modalities—vision and hearing—evoke similar, distributed patterns of frontoparietal activity. A subsequent attentional blink study, using concurrent EEG/MEG, then examined behavior and electromagnetic activity for T1 + T2 pairs, either in the same modality (both auditory or both visual) or different modalities (T1 auditory, T2 visual).

## MATERIALS AND METHODS

### Participants

For both fMRI and MEG/EEG experiments, participants were recruited from the MRC Cognition and Brain Sciences Unit volunteer panel. Twenty-two people were tested for the fMRI study. Two of these were excluded because they did not complete the task, two were excluded because of a technical problem in collecting the responses, and one was excluded due to excessive motion (frequently more than 3 mm). Of the remaining 17 participants, 10 were female and ages ranged from 18 to 39 yr (mean 25 yr). A separate group of 19 people took part in the EEG/MEG experiment, of whom one was not analyzed due to a technical failure in data acquisition. Of the rest, 14 were female, with ages ranging from 20 to 48 yr (mean 26 yr).

All participants reported normal or corrected-to-normal visual acuity, normal hearing, and no history of psychological or neurological impairment. All participants gave their written informed consent and were paid for taking part. Ethical approval was obtained from Cambridge Psychology Research Ethics Committee (CPREC).

### Experiment 1: fMRI

#### Materials, stimuli, and paradigm.

Participants were asked to identify targets presented in streams of auditory and/or visual events. In both auditory and visual streams, targets were letters D or P, and nontargets were other letters (L, M, N, or X). Either 0 (baseline trials), 1, or 2 targets were presented on each trial. For comparability with *experiment 2*, and to check on behavioral data, *experiment 1* used a full attentional blink design. As regards fMRI data, however, our only aim was to estimate separate brain responses to auditory and visual targets. Accordingly we focus just on responses to single targets (contrast of 1-target to baseline trials), avoiding the limits of estimating separate BOLD responses to two targets presented in rapid succession.

**Fig. 1.**
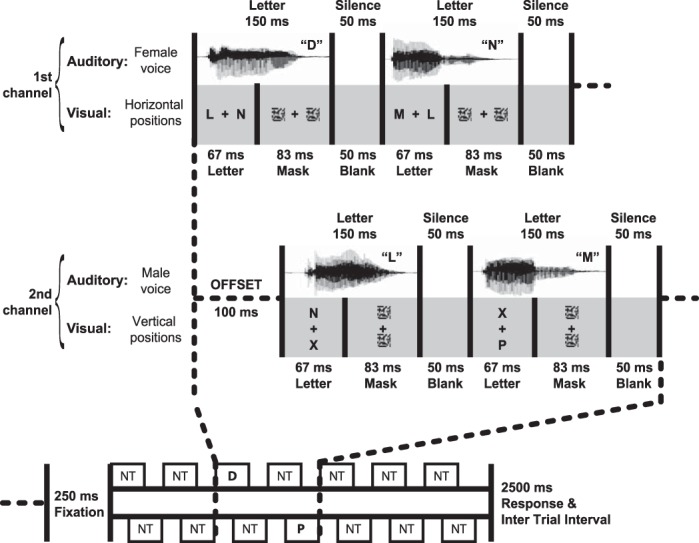
Stimulus streams from *experiment 1*. Stimuli were organized into 2 channels, each consisting of 2 rapid serial visual presentation (RSVP) streams and 1 rapid serial auditory presentation (RSAP) stream of letters. In each session, participants focused attention on just one component of each channel (e.g., auditory component of first channel, visual component of second channel), with T1 and T2 in the attended component of first and second channels, respectively. Each stream contained 7 letters, with T1 in the 3rd position. T2 could follow T1 at stimulus onset asynchronies (SOAs) of 100 ms, 300 ms (illustrated), or 700 ms. “NT” indicates events containing only nontargets (letters LMNX), while “P” and “D” indicate events containing the respective target.

All auditory and visual stimuli were presented using Visual Basic.NET. Visual stimuli were back-projected onto a screen using a Christie video projector with a 60-Hz refresh rate; letters had a visual angle of ∼0.65° and were positioned 1.6° from fixation. Auditory stimuli were presented over pneumatic tube earphones, at a volume audible and comfortable for each participant. Stimulus streams are illustrated in Fig. 1. Stimuli were organized into two channels, each consisting of two rapid serial visual presentation (RSVP) streams of letters and one rapid serial auditory presentation (RSAP) stream of spoken letters. Following previous studies (e.g., Duncan et al. 1997), two visual streams were used in each channel rather than one to encourage central fixation even if only one visual channel was attended. In one channel, the RSAP stream was spoken in a female voice (Fig. 1) while in the other it was spoken in a male voice. Both streams were presented binaurally. For half the participants (as illustrated) the first channel consisted of the female voice RSAP, and RSVPs on the horizontal axis, while the second channel consisted of the male voice RSAP, and RSVPs on the vertical axis; for the other participants, the channels were inverted. Note that we term a target occurring in the first channel T1 and in the second channel T2, even on single-target trials.

Each trial started with a central fixation cross; 250 ms later, stimuli were presented within the first channel as follows: letters appeared in both visual streams for 67 ms (4 frames), followed by a scrambled pattern mask lasting 83 ms (5 frames). A spoken letter of duration 150 ms occurred simultaneously with the written letters. A 50 ms (3 frame) blank screen then preceded the next set of letters. Stimuli in the second channel were presented similarly, but offset by 100 ms to create maximum asynchrony between the channels. Each stream consisted of seven letters and lasted 1,500 ms. There were six equally frequent trial types: no-target (streams with neither T1 nor T2), T1-alone (streams with T1 but no T2), T2-alone, and T1 + T2 with stimulus onset asynchronies (SOAs) of 100, 300, or 700 ms between the two targets. When T1 occurred, it was presented in the first channel 400 ms from the beginning (3rd letter in the stream). When T2 occurred, it was presented on the second channel in the third, fourth, or sixth position, giving SOAs of 100, 300, or 700 ms in T1 + T2 trials. Only the third position was used for T2-alone trials. Participants had 2.5 s from the end of the stream to respond before the task proceeded to the next trial. The order in which responses had to be given was prompted by the program and corresponded to the target presentation order: Participants were requested to respond first to T1 and then to T2, reporting “P,” “D,” or “absent” using a button box.

Participants completed two sessions, one same-modality (T1 and T2 both auditory) and one different-modality (T1 auditory, T2 visual). In the same-modality session, T1 appeared in the auditory stream of the first channel (e.g., Fig. 1, female voice), and T2 in the auditory stream of the second channel (e.g., Fig. 1, male voice). In the different-modality session, T1 again appeared in the auditory stream of the first channel (e.g., Fig. 1, female voice), while T2 appeared in one of the two visual streams in the second channel (e.g., Fig. 1, vertical positions). Thus participants could focus attention just on streams where targets were known to appear; no targets ever appeared in other streams.

Sessions were run consecutively in the scanner, with order counterbalanced across participants. Each session contained four blocks, separated by 15 s of fixation. Within each block, there were 16 repetitions of each of the six different trial types, presented in random order.

Prior to the experiment, participants received a short practice. The practice was organized in blocks of 16 trials each. The first block was a practice in performing the discrimination task on T1. This block was repeated as necessary until the participant reached at least 70% accuracy, at which point they moved onto the second phase of practice which focused on correct identification of T2. Again, participants continued until they reached at least 70% accuracy. The last practice block consisted of a mixture of the six trial types presented in the main experiment.

#### Recording and analysis.

Images were acquired on a Siemens 3T Tim Trio scanner (Siemens Medical Systems, Erlangen, Germany). A T1-weighted structural image for each participant used an MPRAGE sequence (TR = 2,250 ms, TE = 2.99 ms, TI = 900 ms, flip angle = 9°, matrix 256 × 240 × 192, voxel size = 1 mm × 1 mm × 1 mm). To record BOLD signal during the task, a quiet EPI sequence was adopted (Peelle et al. 2010; Schmitter et al. 2008), which uses a sinusoidal readout echo train to reduce acoustic scanner noise produced by gradient coil switching. We acquired 32 slices in descending order, with a 0.75-mm gap between slices (TR = 2.64 s, TE = 44 ms, flip angle = 83° FOV = 192 × 192 mm, matrix = 64 × 64, voxel size = 3 × 3 × 3 mm).

Image preprocessing and statistical analyses were performed using SPM5 (Wellcome Trust Centre for Neuroimaging, UCL, London; http://www.fil.ion.ucl.ac.uk/spm), Matlab (The MathWorks), and “automatic analysis” software (Cusack et al. 2015). The first five images per session were discarded. Images for each participant were realigned to the first image in the series, slice time-corrected, coregistered with the structural image, and normalized to MNI152 space. Prior to statistical analysis the data were spatially smoothed with a 10-mm FWHM isotropic Gaussian kernel.

Data were initially analyzed separately for each participant, using a general linear model. Low-frequency drifts were removed with high-pass filtering (with a cutoff period of 128 s) and autocorrelations were modeled using a first-order autoregressive model. Each event of interest (trial type) was modeled from onset to offset of the stimulus streams, convolved with a standard hemodynamic response function to create the regressors used in the model. The six motion parameters obtained during realignment were included in the model as additional regressors. Following analysis on the participant level, images containing the contrasts of parameter estimates for each participant were entered into second-level random-effects analyses. Initial contrasts tested responses to single auditory targets (T1-alone or T2-alone) vs. no-target trials, and single visual targets (T2-alone) vs. no-target trials, combining both sessions to maximize power. Further analyses directly contrasted responses to auditory T2-alone vs. responses to visual T2-alone, and tested for a significant response to both auditory and visual T2 alone. These latter analyses defined single-target responses using T2-alone trials vs. no-target trials from their respective sessions, so that the number of trials was balanced across auditory and visual modalities, and the contrasts entering the conjunction analysis used independent data.

### Experiment 2: EEG/MEG

#### Materials, stimuli, and paradigm.

In the EEG/MEG experiment, events were similar except that each stream consisted of 12 letters and lasted 2,700 ms; T1, if present, was the fourth letter in the first channel, while T2 was the fourth, fifth, or seventh in the second channel (always 4th on T2-alone trials). In this study we estimated separate electromagnetic responses to all targets, on both single-target (T1-alone or T2-alone) and T1 + T2 trials. Stimuli were back-projected onto a screen at a refresh rate of 60 Hz, using a Panasonic DLP projector; letters had a visual angle of ∼0.84° and were positioned 2.1° from fixation. The background was midgray, with a small black central cross to mark the fixation point. Sound stimulation was delivered at a comfortable volume using Etymotic ER3-14A air ear phones.

Each volunteer completed three sessions, two of them same-modality (both targets auditory or both visual) and one different-modality (T1 auditory, T2 visual). The three sessions took place on different days, separated by ∼7 days. Session order was counterbalanced across participants. In each session, each trial type was repeated 80 times, totaling 480 trials, which were split into five blocks of ∼10 min each. Trial types were randomly mixed. In this study, separate prompts at the end of each trial required a two-alternative forced-choice response (D or P?) for each target presented; no response was requested for targets not presented. The program proceeded once responses were given. As in the fMRI study, participants completed a practice session before starting the main experiment.

#### Recording and analysis procedure.

Magnetic fields were measured using an Elekta Neuromag VectorView MEG system (Stockholm/Helsinki). Participants were tested in a sound- and magnetically-shielded room to prevent contamination from noncerebral magnetic signals. Volunteers were seated, and responded using a pair of MEG-compatible button boxes held in either hand. Electromagnetic signals were sampled at 1 kHz, with a high-pass filter cut-off at 0.03 Hz. The times of stimulus events and responses were recorded as impulses on a trigger channel.

At the beginning of each block, the position of the head relative to the MEG helmet was measured using four or five “Head Position Indicator” (HPI) coils attached to the EEG cap, whose positions had previously been localized relative to anatomical landmarks (nasion and periauricular points) using a 3D digitizer (Fastrak Polhemus, Colchester, VA). A pair of electrodes was positioned above and below the left eye to measure blinks and vertical eye-movements; a pair of electrodes at the temples monitored horizontal eye-movements. EEG was acquired concurrently to the MEG signal using an elastic cap from Elekta Neuromag for the first 8 participants, and an elastic cap from Easy Cap for the last 10 participants. In both cases, 70 Ag/AgCl electrodes were mounted in the cap, including the international 10–20 system sites plus 51 electrodes interspersed according to the 10-10 system. The reference electrode was placed on the tip of the nose. Impedance at each electrode site was maintained lower than 5 kΩ.

An overview of the analysis strategy is given in Table 1. Maxfilter 2.0 (Elekta Neuromag, Helsinki) was initially used to preprocess the MEG data using “Signal Space Separation” (Taulu et al. 2005). Bad channels were automatically detected and removed by the software, and data were reconstructed eliminating field patterns produced by noise sources located outside the sensor array. During this preprocessing stage, the head position of each participant was realigned to the average position of all participants in order to compensate for head movement during the recordings.

**Table 1.** Analysis of event-related potential/field components: summary of processing pathway

	Analysis Step	Description
*1*	Preprocessing	Temporal filtering, artefact removal, epoching, baseline subtraction, bad channel/epoch rejection, averaging
*2*	Create target-related difference waves	(T1-alone) − (No target)
		(T2-alone) − (No target)
		(T1 + T2) − (T1-alone), per SOA
		Aligned on target onset
*3*	Average single-target responses per modality	Mean of all single-target difference waves, combined from same-modality and different-modality sessions
*4*	Grand average target response per modality	Equal weighting for mean single-target response, and for (T1 + T2) − (T1-alone) at each SOA
*5*	Threshold grand average to create COI per modality	Group analysis for significant target response versus baseline; separately for EEG positivity and negativity, and magnetometers
*6*	Extract individual target-related responses from COIs	Per subject, per condition (mean single-target response, and (T1 + T2) − (T1-alone) at each SOA/session); mean across COI for EEG; RMS across COI for magnetometers
*7*	Statistical comparisons	Planned paired *t*-tests (each SOA vs. single-target response) and repeated-measures ANOVAs

SOA, stimulus onset asynchrony; COI, component of interest.

Subsequent analysis steps for EEG and MEG were performed using SPM5 (Wellcome Trust Centre for Neuroimaging, UCL, London; http://www.fil.ion.ucl.ac.uk/spm), Matlab (The MathWorks), and “automatic analysis” software (Cusack et al. 2015). Continuous data were low-pass and high-pass filtered (with cut-offs at 40 Hz and 1 Hz, respectively), using a fifth-order Butterworth filter in both forward and reverse directions. Independent components analysis (ICA), implemented using EEGLAB (Delorme and Makeig 2004), was used to automatically detect and remove artefactual temporal components correlated with eye movements. Components were projected out of the data if their temporal correlation with either EOG channel exceeded 0.3, or had a spatiotemporal profile indicative of a pulse artifact.

Epochs were extracted around targets of interest, spanning −100 ms to 800 ms relative to target onset. For construction of difference waves (see below), equivalent subtraction epochs (epochs when targets would appear on other trials) were also constructed. For no-target trials there were two such epochs, corresponding to the times of targets in T1-alone and T2-alone trials. For T1-alone trials, there were three subtraction epochs, corresponding to the times at which T2 could appear on T1 + T2 trials. For each time point and each sensor, epochs were baseline-corrected by subtracting the mean signal across 100 ms preceding the time at which T1 could occur. Epochs from the five blocks of each session were then concatenated for each participant.

For EEG, bad channels and epochs were identified and rejected by thresholding. Epochs were rejected if the signal range exceeded 180 μV peak to peak. Channels were marked as bad if more than 15% of epochs met these criteria. For both EEG and MEG, further epochs were rejected if blinks were observed on the basis of large vertical EOG signal, i.e., if mean or maximum signal magnitude over the interval 0–800 ms exceeded criteria based on the signal distribution over the interval −100 to 0 ms across trials (5 SDs for mean magnitude, 7 SDs for maximum). The percentage of trials rejected for EEG across participants and sessions ranged between 0 and 24%, while for MEG it ranged between 0 and 19%.

EEG signal was analyzed keeping the original reference used during the recordings. ERP waveforms were computed by averaging epochs of each type. Difference waves were then constructed to obtain isolated evoked responses to targets of interest: The corresponding subtraction epochs from no-target trials were subtracted from T1-alone and T2-alone epochs, while subtraction epochs from T1-alone trials were subtracted from T2 epochs on T1 + T2 trials. These event-related difference waves were converted into 3D spatiotemporal volumes by stacking their topographies along peristimulus time.

To analyze the EEG signal, we first defined components of interest (COIs) as regions within the space-time volume that showed significant responses to targets (analogous to functional regions of interest in fMRI). Separate COIs were derived for auditory and visual targets. In the first step for each modality, a mean single-target response was derived by averaging responses to the two single targets (T1-alone and T2-alone) from the same-modality session, along with the corresponding target (T1- or T2-alone) from the different-modality session. For auditory targets, a grand average was then obtained by averaging this mean single-target response with separate T2 responses derived from each kind of dual-target trial (estimated T2 responses from T1 + T2 trials at each SOA in the auditory-auditory session). For visual targets, the procedure was identical, except that the mean single-target response was averaged with six dual-target trial T2 responses (separately derived for each SOA in both visual-visual and auditory-visual sessions). In this way, the grand average derived for each modality equally weighted single-target trials and T2 responses for each kind of dual-target trial. Spatiotemporal components of interest (COIs) were then defined based on these grand average responses, where there was a 0.99 posterior probability of the signal exceeding two standard deviations of the mean absolute signal in the 100 ms preceding the target (Friston et al. 2002). For each modality, regions of the EEG space-time volume with negative responses were clearly clustered together into one early COI, and regions with positive responses formed a second, later COI. For each EEG COI, we extracted the mean amplitude for each condition of interest (single-target baseline, and T2 at each SOA).

A similar procedure was used for MEG. Since the topography across the magnetometers had both positive and negative lobes at each time point, a single MEG COI was defined per modality, disregarding the sign of the deflection. As a measure of target-related MEG activity, we took the RMS across all spatial locations and time points within these COIs, again after subtraction of matched epochs without the target of interest.

For both EEG and MEG, extracted values were submitted to paired-sample *t*-tests at the group level to assess differences between the single-target baseline and the T2 response at each SOA. Repeated-measures analyses of variance were also performed on the responses to visual T2, with factors of SOA (100, 300, 700 ms) and session (auditory-visual, visual-visual). For comparisons using a within-session T2-only baseline (see results), the same principles were employed. Again, auditory and visual COIs were derived from grand averages that gave equal weight to T2 responses separately estimated for each type of dual-target trial, along with comparison T2-alone responses.

We note that comparing two estimated T2 responses—one obtained by subtracting no-target from T2-alone, the other by subtracting T1-alone from T1 + T2—is formally a test for additivity of the T1 and T2 responses on the T1 + T2 trial. Following usual practice (see, e.g., Ptito et al. 2008; Valdes-Sosa et al. 2004), we interpret any difference between the two subtractions in terms of changed T2 response, but a change in T1 response for T1-alone and T1 + T2 trials will also affect the [(T1 + T2) − T1-alone] subtraction. Accordingly, data from an attentional blink design are ambiguous in distinguishing altered T2 response from altered T1 response; for present purposes, however, the key point is that equal T2 estimates from the two subtractions imply perfect additivity of T1 and T2 responses.

## RESULTS

### Experiment 1: fMRI

**Fig. 2.**
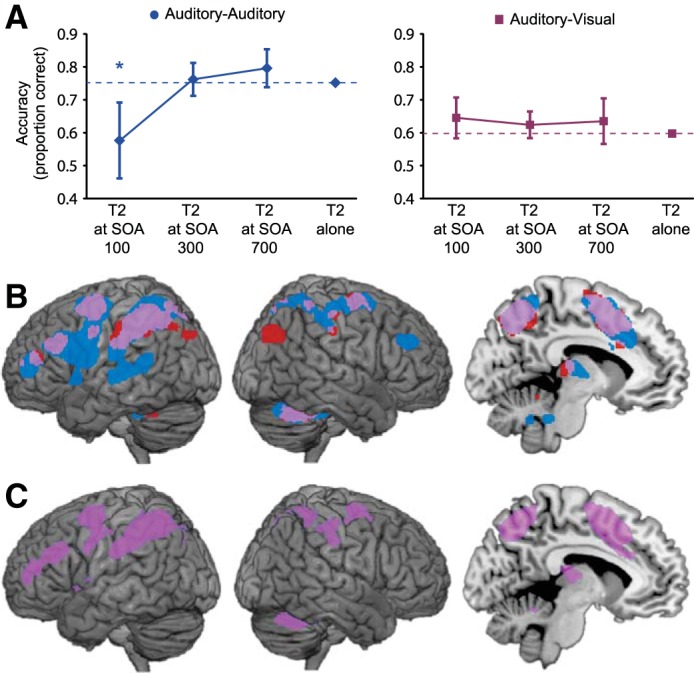
*Experiment 1*. *A*: behavioral results. Separate planned *t*-tests within each session compared the identification accuracy for T2, at each SOA following T1, with the accuracy for T2 when not preceded by T1. *P < 0.01 (2-tailed); error bars are 95% confidence intervals for the difference from single target accuracy. *B*: fMRI responses to single auditory and visual targets, relative to nontarget trials. Significant activations are shown at *P* < 0.05, corrected for multiple comparisons using the false discovery rate (FDR). Responses to auditory and visual targets are shown in blue and red, respectively, with overlap in purple. *C*: a conjunction analysis, identifying regions that respond significantly to auditory and visual T2-alone trials, confirms the overlap above (purple; *P* < 0.05, FDR).

Behavioral data from *experiment 1* are shown in Fig. 2*A*. The presence of an attentional blink was assessed by separate *t*-tests that compared the accuracy of T2 identification at each SOA with accuracy when T2 was presented without a preceding T1. We found a strong attentional blink in the unimodal (auditory-auditory) session, with T2 discrimination significantly impaired at SOA 100 and then recovering as SOA increased [Fig. 2*A*, *left*; SOA 100: *t*(16) = 2.80, *P* < 0.01; SOA 300: *t*(16) = 0.49, *P* > 0.1; SOA 700: *t*(16) = 1.14, *P* > 0.1]. In contrast, when the two targets were presented in different modalities (auditory-visual session) no significant attentional blink was found at any SOA [Fig. 2*A*, *right*; SOA 100: *t*(16) = 1.16; SOA 300: *t*(16) = 0.88; SOA 700: *t*(16) = 0.59; all *P* > 0.1].

Our prime use of *experiment 1* was to measure BOLD activity in response to single targets in auditory and visual modalities. For this purpose, we used contrasts of single target (T1-alone or T2-alone) vs. no-target trials. For auditory targets, data were available from T1- and T2-alone trials in the auditory-auditory session, as well as T1-alone trials from the auditory-visual session; all these were combined to estimate the response. For visual targets, data were available just from T2-alone trials in the auditory-visual session. Results are shown in Fig. 2*B*. Activity was stronger for the auditory case, which had more data, but for both modalities, there was activity in lateral and medial parietal cortex, lateral frontal cortex, and within the presupplementary motor area, all characteristic regions of the global workspace (Dehaene and Naccache 2001; Dehaene et al. 2003). Except for a small region of right occipitoparietal cortex, the active region for visual targets was almost entirely included in the larger region for auditory targets. For auditory targets only, there was additional activity in modality-specific auditory cortex. In line with the global workspace model, the results show extensive overlap in frontoparietal regions recruited by different kinds of behaviorally-significant events. A similar pattern was seen when directly testing for a conjunction between the responses to auditory targets and visual targets (Fig. 2*C*), using just T2-alone vs. no-target contrasts to make data for the two modalities independent (Heller et al. 2007). Directly contrasting responses to T2-alone in the two modalities did not reveal any activations that were significantly greater for one modality than the other.

### Experiment 2: EEG/MEG

**Fig. 3.**
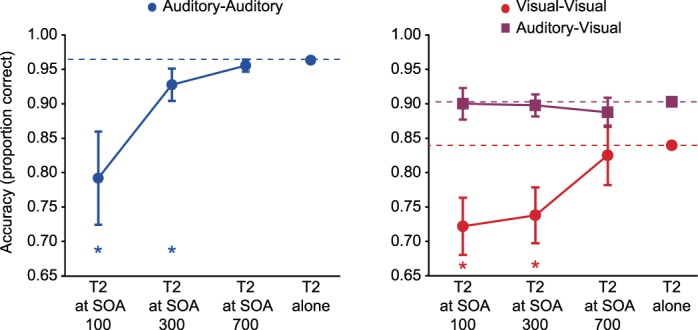
*Experiment 2*: behavioral results. Separate planned *t*-tests within each session compared the identification accuracy for T2, at each SOA following T1, with the accuracy for T2 when not preceded by T1. *P < 0.01 (2-tailed); error bars are 95% confidence intervals for the difference from single target accuracy.

Behavioral data from *experiment 2* are shown in Fig. 3. The presence of an attentional blink was assessed by separate *t*-tests that compared the accuracy of T2 identification at each SOA with accuracy when T2 was presented without a preceding T1. We found a strong attentional blink effect within the same modality, with T2 discrimination significantly impaired at SOAs 100 and 300 in both the auditory-auditory session [Fig. 3, *left*; SOA 100: *t*(17) = 5.01, *P* < 0.01; SOA 300: *t*(17) = 3.09, *P* < 0.01] and the visual-visual session [Fig. 3, *right*, red circles; SOA 100: *t*(17)=5.54, *P* < 0.01; SOA 300, *t*(17) = 4.93, *P* < 0.01]. In both modalities, as usual, T2 discrimination performance had recovered by SOA 700. In contrast, when the two targets were presented in different modalities (auditory-visual session) no significant attentional blink was found at any SOA (Fig. 3, *right*, purple squares). Dual-target interference was also observed on the responses to T1, although of smaller magnitude than for T2, and again only in the unimodal conditions: Accuracy was never reduced by more than 3% of T1-alone accuracy, except for SOA 100 in the auditory-auditory session [13% reduction, *t*(17) = 5.50, *P* < 0.01] and SOA 100 in the visual-visual session [5% reduction, *t*(17) = 3.52, *P* < 0.01].

**Fig. 4.**
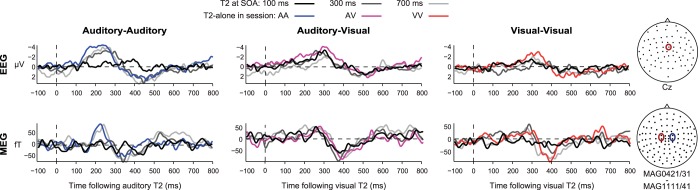
*Experiment 2*: example time courses of event-related potentials (ERPs)/fields (ERFs) in response to T2 onset for the various SOAs in each condition. ERPs are illustrated for electrode Cz; ERFs show the average of the highlighted magnetometers, after negating the signal on the right-hand side to account for the antisymmetry of the fields.

**Fig. 5.**
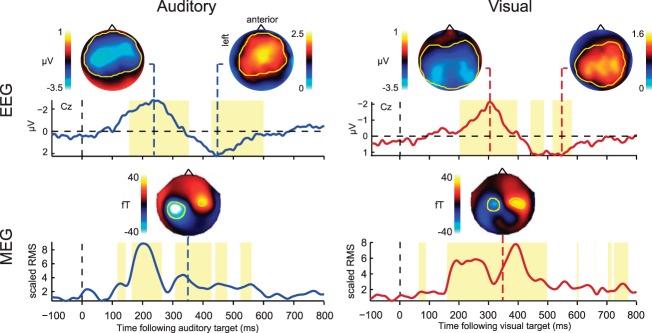
*Experiment 2*: grand average EEG and MEG responses to auditory and visual targets, averaging estimates from single-target trials (T1-alone and T2-alone, each with no-target trials subtracted) and dual-target trials (T1 + T2 at each SOA, each with T1-alone trials subtracted; see materials and methods). The EEG time course is illustrated for electrode Cz; topographies are plotted at negative and positive peaks (236 and 444 ms, respectively, following auditory target onset, and 300 and 544 ms following visual target onset). Overlaid yellow contours indicate significant difference from the baseline period, defining spatiotemporal components of interest (COI) for subsequent analyses; yellow time windows illustrate when these components were significant at any electrode, reflecting early (negative) and late (positive) EEG deflections for each sensory modality. The MEG time course shows RMS across all magnetometers within the COI, divided by the mean pretarget response. Topographies show the signal across the magnetometers at 348 ms following target onset.

To analyze the electrophysiological signal, difference waves were created to isolate the response to individual targets. For single-target trials, the activity on no-target trials was subtracted; for dual-target (T1 + T2) trials at each SOA, the amplitude of the electric and magnetic response to T2 was calculated by subtracting mean activity in T1-only trials from mean activity in T1 + T2 trials, and aligning the resulting difference wave to the onset of T2. Example difference waves reflecting T2 responses are illustrated in Fig. 4 for the different SOAs and sessions. For each modality, we also derived an average single-target baseline, obtained by averaging the single-target difference waves, aligned to target onset, across the three types of single targets in each modality (auditory: T1-alone and T2-alone from the auditory-auditory session, along with T1-alone from the auditory-visual session; visual: T1-alone and T2-alone from the visual-visual session, along with T2-alone from the auditory-visual session). To summarize the electrophysiological signal, spatiotemporal COIs were then defined based on significant responses to targets, separately for the two modalities. COIs were obtained from a grand average target response in each modality, equally weighting the single-target baseline response and the T2 response from T1 + T2 trials at each SOA (see materials and methods). In each modality, this procedure identified an earlier EEG negativity followed by a later positivity (Fig. 5, *top*), resembling previously-reported N2-P3 components with relatively broad topographies. For each condition, mean EEG amplitude was extracted from the spatiotemporal target-responsive COIs. As a measure of target-related MEG activity, we took the root-mean-square (RMS) across the COI (Fig. 5, *bottom*).

**Fig. 6.**
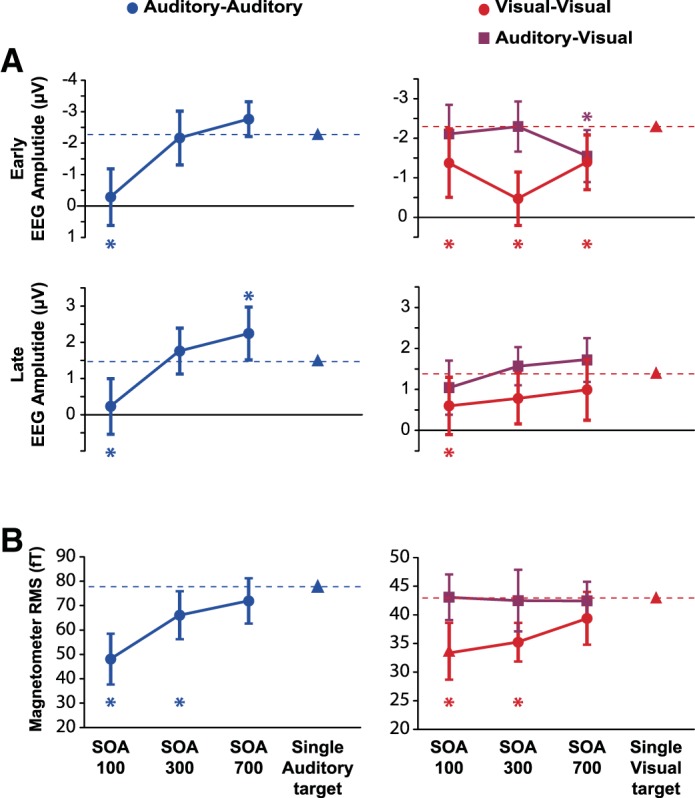
*Experiment 2*: EEG (*A*) and MEG (*B*) responses. Separate planned *t*-tests, within each session and each spatiotemporal COI, compared response amplitude to T2 at each SOA (T1 + T2 trials) with response amplitude to a single target of the corresponding modality. *P < 0.05 (2-tailed); error bars are 95% confidence intervals for the difference from the single-target response.

The extracted electrophysiological signals were then analyzed in a similar way to the behavior, by *t*-tests comparing T2 responses derived from T1 + T2 trials to the single-target baseline. In EEG data from the auditory-auditory session (Fig. 6*A*, *left*), findings for both the early negativity and later positivity strongly resembled behavioral data, with a significant reduction of early negativity [*t*(17) = 4.67; *P* < 0.001] and late positivity [*t*(17) = 3.47; *P* < 0.005] at SOA 100. The late positivity was significantly larger than baseline at SOA 700 [*t*(17) = 2.16, *P* < 0.05]. Data from the visual-visual session (Fig. 6*A*, *right*, red circles) also resembled behavioral data, with a significant reduction of the early negativity at SOA 100 [*t*(17) = 2.25; *P* < 0.05], SOA 300 [*t*(17) = 5.69; *P* < 0.001], and SOA 700 [*t*(17) = 2.74; *P* < 0.05], together with a significant reduction in late positivity just at SOA 100 [*t*(17) = 2.24; *P* < 0.05]. In the auditory-visual session, in contrast, both early negativity and late positivity for the visual T2 were indistinguishable from single-target values at SOAs 100 and 300 (Fig. 6*A*, right, purple squares). The only difference from baseline was the early negativity at SOA 700 [*t*(17) = 2.38, *P* < 0.05]. In line with prediction, the results show largely preserved ERPs for a visual T2 presented immediately following an auditory T1. Thus responses to T1 and T2 were additive in the auditory-visual session: Across SOAs, the subtraction of activity on T1-only trials from T1 + T2 trials produced the same difference ERP as the subtraction of no-target from single-target trials.

RMS of the MEG signal showed similar results (Fig. 6*B*). In the auditory-auditory session, response to T2 was significantly reduced, both for SOA 100 [*t*(17) = 5.77; *P* < 0.001] and SOA 300 [*t*(17) = 2.40; *P* < 0.05]. In the visual-visual session, response to T2 was also reduced for SOA 100 [*t*(17) = 3.43; *P* < 0.01] and SOA 300 [*t*(17) = 3.15; *P* < 0.01]. Again, however, magnetic responses to T2 in the auditory-visual session were indistinguishable from responses to single targets.

For maximum stability, the above analyses used an average single-target baseline for each modality, obtained by combining data from T1-alone and T2-alone trials in both same-modality and different-modality sessions. Differences between T1-alone and T2-alone responses, and between same-modality and different-modality sessions, were inconsistent across early/late time windows and across EEG/MEG. In a follow-up analysis, EEG/MEG analyses were repeated comparing the T2 response on dual-target trials not to the average single-target baseline as above, but to the T2-only response from the corresponding session. Results were largely unchanged, except that, in EEG data from the auditory-visual session, reduction of early negativity on T1 + T2 trials was seen not just at the longest SOA, as in the main analysis, but across all SOAs.

Importantly, a final set of analyses directly compared visual T2s in visual-visual and auditory-visual sessions, using analysis of variance with factors of session and SOA. For behavior (Fig. 3), there was a significant main effect of session [*F*(1,17) = 43.1, *P* < 0.001], and an interaction with SOA [*F*(2,34) = 12.4, *P* < 0.001]. For EEG/MEG (Fig. 6), all COIs showed a main effect of session [early EEG: *F*(1,17) = 14.5, *P* < 0.005; late EEG: *F*(1,17) = 10.3, *P* < 0.01; MEG: *F*(1,17) = 14.6, *P* < 0.005], interactions were significant for the early EEG negativity [*F*(2,34) = 5.00, *P* < 0.05], and marginal for MEG [*F*(2,34) = 2.51, *P* = 0.096].

## DISCUSSION

When two target events occur in rapid succession, the well-known attentional blink suggests strong competition for processing resources. In particular, detection or identification of the second target suffers severe interference if the interval between the two is much less than 0.5 s (Raymond et al. 1992). In line with reduced accuracy on the second target, there is suppression of the typical EEG signature of target processing, the N2-P3 complex (Sergent et al. 2005; Vogel et al. 1998). An influential proposal relates these phenomena to processing limits in a frontoparietal global workspace, underlying widespread restrictions on concurrent attention and awareness (Sergent et al. 2005).

Dividing attention, however, is often much easier if concurrent tasks are dissimilar, e.g., involving stimuli (Treisman and Davies 1973) or responses (McLeod 1978) in different modalities. Such findings question universal processing competition in a global frontoparietal workspace. With targets in different sensory modalities, in particular, the attentional blink can be reduced or completely absent (Duncan et al. 1997; Hein et al. 2006; Soto-Faraco and Spence 2002). Here we used fMRI, EEG, and MEG to examine the attentional blink with targets in same or different modalities.

First, we used fMRI to examine brain regions responsive to targets. Using single auditory and visual targets, we found extensive activity in frontal and parietal regions usually associated with the global workspace. In these regions, furthermore, there was substantial overlap in activity for targets in the two modalities. Such results match previous findings of extensive frontoparietal activity for targets and other behaviorally significant events of many different kinds (e.g., Hampshire et al. 2010; Hon et al. 2006; Jiang et al. 2000).

To examine the attentional blink, we used trials with two successive targets, presented at varying SOA. Now we moved from fMRI to EEG/MEG, exploiting the higher temporal resolution of EEG/MEG data. In same-modality conditions (visual-visual or auditory-auditory), results were in line with previous findings (Sergent et al. 2005; Vogel et al. 1998), and with predictions from the global workspace theory. Behavioral data showed a typical attentional blink, along with a reduced N2-P3 complex in EEG, and reduced target-related activity in MEG. For auditory-auditory trials, electromagnetic responses closely paralleled behavior, with reduced responses at short SOA and recovery at long SOA; for visual-visual trials the trends were similar, although some reduction of electromagnetic responses remained even at the longest SOA. Results were very different when T1 was auditory and T2 visual. In behavior, all trace of an attentional blink disappeared; even at the shortest SOA, targets were identified without mutual interference. Correspondingly, electromagnetic responses to the two targets were largely additive, indicating parallel brain responses. The one deviation from additivity was reduction of the early negative component of the visual T2 response; even this reduction, however, appeared only in EEG, not MEG data, and unlike an attentional blink, was most pronounced at the longest SOA.

N2-P3 and MEG responses had diffuse scalp topographies (Fig. 5), and as usual in EEG/MEG data, their sources are uncertain. In addition to overlapping activity in frontoparietal cortex, our fMRI data showed some regions of separate activity for targets in different modalities, in particular a selective region of activity in auditory cortex seen only for auditory targets, although these did not reach significance in a direct contrast of T2 responses in the two modalities. In principle, modality-specific regions could make some contribution to target-selective responses recorded with EEG/MEG. It is also possible that EEG/MEG responses could receive some contribution from additional regions not revealed by our fMRI data. These possibilities notwithstanding, it seems likely that N2-P3 responses were at least partly generated in the extensive frontoparietal regions of target-selective activity shown by fMRI. This would be consistent with previous attempts at source localization for these components (Bledowski et al. 2004; Sergent et al. 2005), and with the proposals of global workspace theory (Sergent et al. 2005; Sergent and Dehaene 2004). On this interpretation, our data suggest that, when simultaneous target events are dissimilar, there is some capability for parallel frontoparietal responses, reflecting reduced susceptibility to competition, cross-talk, or mutual interference. For example, much evidence shows flexibility in frontoparietal activity, with neurons adapting their properties to code currently attended information (Duncan 2010, 2013; Kadohisa et al. 2013; Rigotti et al. 2010). In this process, one possibility is that somewhat separate pools of neurons may be dedicated to very dissimilar representations; consistent with this, multivoxel pattern analysis of fMRI data suggests discriminable patterns of activity for visual and auditory events in frontoparietal cortex (Tamber-Rosenau et al. 2013). More work is needed, however, to tie the parallel EEG/MEG responses we observed to specific neural generators.

As noted earlier, large attentional blinks can sometimes be observed even for targets in different sensory modalities. Although it is uncertain what task conditions are critical, factors encouraging strong interference may include speeded responses to T1 (Jolicoeur 1999a; Jolicoeur 1999b), changes in task demand between T1 and T2 (Arnell and Jolicoeur 1999; Jolicoeur 1999b), and increasing T1 task demand (Arnell and Duncan 2002). When a between-modality blink occurs, furthermore, EEG data match behavior in showing suppressed T2 responses (Arnell 2006; Ptito et al. 2008). Evidently, stimuli in different sensory modalities are not protected from mutual interference under all circumstances. Such interference is often minimal, however, for simple, unspeeded tasks, and under these circumstances, our data show parallel, largely independent neural responses.

The core idea of global workspace theory, that simultaneous events compete for representation in a distributed frontoparietal network, fits broad behavioral limits on attentional capacity and dual task performance (Bourke et al. 1996; Kahneman 1973). It is also consistent with fMRI (Marois and Ivanoff 2005) and single unit (Kadohisa et al. 2013; Watanabe and Funahashi 2014) data linking limited attentional capacity to frontoparietal activity. At the same time, behavioral data reveal remarkable cases of parallel processing, especially when simultaneous tasks involve very different operations and content (Allport et al. 1972; Duncan et al. 1997; McLeod 1978). Under these circumstances, correspondingly, the brain's ability for parallel processing may extend to the frontal and parietal network proposed to make up a global neuronal workspace.

## GRANTS

This work was supported by MRC intramural program MC-A060-5PQ10 and MC-A060-53144, a grant for women in research from the University of Chieti-Pescara “G. D'Annunzio,” and a grant for research abroad from the University of Rome “La Sapienza.”

## DISCLOSURES

No conflicts of interest, financial or otherwise, are declared by the author(s).

## AUTHOR CONTRIBUTIONS

Author contributions: P.F., V.P., and J.D. conception and design of research; P.F. and C.B. performed experiments; P.F., D.J.M., and O.H. analyzed data; P.F., D.J.M., and J.D. interpreted results of experiments; P.F. and D.J.M. prepared figures; P.F. and J.D. drafted manuscript; P.F., D.J.M., and J.D. edited and revised manuscript; P.F., D.J.M., O.H., C.B., V.P., and J.D. approved final version of manuscript.
